# Estimation of liver standardized uptake value in F18-FDG PET/CT scanning: impact of different malignancies, blood glucose level, body weight normalization, and imaging systems

**DOI:** 10.1007/s12149-024-01985-7

**Published:** 2024-10-16

**Authors:** Mohamed S. Abd-Elkader, Sherif M. Elmaghraby, Mohamed A. Abdel-Mohsen, Magdy M. Khalil

**Affiliations:** 1 Department of Physics, Faculty of Science, Helwan University, Cairo, Egypt; 2 Nuclear Medicine and Radiation Oncology Department (NEMROK), Cairo University Hospitals, Faculty of Medicine, Cairo University, Cairo, Egypt; 3 School of Applied Health Sciences, Badr University in Cairo (BUC), Cairo, Egypt; 4 Medical Biophysics, Department of Physics, Faculty of Science, Helwan University, Cairo, Egypt

**Keywords:** Liver, SUV, Blood glucose, PET/CT, Normalization

## Abstract

**Introduction:**

The aim of this work was to investigate homogeneity and stability of liver SUV in terms of different malignancies considering different body normalization schemes and blood glucose concentrations as well as PET/CT imaging systems.

**Methods:**

The study included 207 patients with four different types of cancers namely breast, lymphoma, lung, and bone-metastasis. Data acquisition was performed with GE Discovery IQ, Biograph mCT, uMI 550, and Ingenuity TF64 after a single intravenous injection of 194 ± 67.5 MBq of 18F-FDG.

**Results:**

In body weight normalization, SUVmax and SUVmean in bone-mets as well as SUVmean in lung patients were not statistically different among scanners especially for data corrected for glucose levels (*p = *0.062, 0.121, and 0.150, respectively). In SUVlbm derived from lung patients, there was no significant differences in Philips in comparison to GE and Siemens (both, *p > *0.05) for data corrected and not corrected for glucose levels. In SUVbsa, the only non-significant difference revealed among scanners was in the measurements of SUVmean obtained from lung and bone-mets (*p = *0.107 and 0.114) both corrected for glucose levels. In SUVbmi, SUVmean of lung and bone-mets as well as SUVmax of bone-mets showed a non-significant differences among the four different scanning systems (*p = *0.303, 0.091, and 0.222, respectively) for data corrected for glucose levels.

**Conclusion:**

Liver glucose correction needs further investigations in individual tumors but could be potentially affected by whether measurements are made on SUVmean versus SUVmax, body weight normalization, as well as the imaging system. As such, selection of normalization to body weight method should be carefully selected before clinical adoption and clinically adopted and body surface area would provide the highest correlation. As such, normalization of body weight should be carefully made before clinical adoption. SUVmean proves to be useful and stable metric when liver is corrected for blood glucose levels.

**Supplementary Information:**

The online version contains supplementary material available at 10.1007/s12149-024-01985-7.

## Introduction

Fluorine-18-fluorodeoxyglucose (^18^F-FDG) positron emission tomography/computed tomography (PET/CT) has become an indispensable tool in the management of malignancy, from staging of diseases and planning treatment to monitoring therapy response [1]. Established procedure guidelines for tumor imaging with ^18^F-FDG PET/CT recommend the use of semi-quantitative metrics for the estimation of tumor glucose metabolism.

SUV is a dimensionless metric used to evaluate tracer uptake on PET imaging. It helps physicians distinguish between normal and abnormal levels of uptake. It is normalized for body weight (BW) and injected dose. If the dose is uniformly distributed over the entire body, the SUV value everywhere in the body would be 1.0

$${\text{SUV }} = { }\frac{{{\text{Concentration of activity in tumor}}\left( {{\text{MBq}}/{\text{mL}}} \right){ }}}{{{\text{Injected activity}}\left( {{\text{MBq}}} \right)/{\text{mass of patient}}\left( {\text{g}} \right)}}$$

SUV is a relative quantitative measurement with many factors impacting the result, such as patient size, time of measurement, dose extravasation, partial volume effects, and reconstruction parameters. To address these challenges, the calculation of SUV can be adapted; for example, with the substitution of body weight by lean body mass (LBM), body surface area (BSA), or body mass index (BMI).

SUVs are of major interest in clinical studies as they can be used to accurately assess tumor response. Standard oncology criteria are often based on tumor size. However, changes in size due to therapy can take months, causing a delay in therapeutic decisions. Change in SUV can occur much earlier, providing an alternative approach to tumor response assessment. PERCIST criteria, for example, assess metabolic response by evaluating SUL (SUV normalized to LBM) at each time point. However, in the context of clinical trials, to ensure the comparability of results across patients and sites, it is crucial to standardize image acquisition.

The normalization of SUV in PET/CT imaging poses several challenges and limitations that need to be addressed. One major challenge is the variability in SUV measurements due to factors, such as patient motion, image noise, and reconstruction algorithms [2–5]

Additionally, SUV normalization assumes that the tissue being measured is metabolically stable over time, which may not always be the case. Moreover, there is a lack of standardization in SUV measurement protocols, hindering inter-study comparisons. Furthermore, certain physiological conditions such as inflammation or altered glucose metabolism can also impact SUV values, further complicating the interpretation of results [4, 6].

There are also several challenges associated with SUV measurements, including inter- and intra-patient variability, scanner differences, and reconstruction algorithms. As a result, various normalization techniques have been developed to improve the accuracy and reliability of SUV measurements. Recent advances have focused on the development of patient-specific SUV normalization methods to account for individual variations in metabolism and body composition [4, 7, 8]

According to guidelines from the European Association of Nuclear Medicine (EANM), tumor SUV should be normalized to blood glucose levels of 5 mmol/L, because the two variables are inversely correlated [1]. The majority of departments would accept blood glucose values up to 10 mmol/L, although EANM recommendations also advise delaying the study if blood glucose is higher than 7 mmol/L.

An alternate method is to express the tumor count rate as a ratio with the liver count rate [4, 6], since it is believed that the liver count rate (SUVliver) is relatively constant [2, 3]. The truthfulness of this strategy has drawn attention lately because of the possible impact of hepatic steatosis on liver FDG uptake. Numerous teams have investigated the connection between SUVliver and steatosis. However, the findings were contradictory [9, 10], perhaps as a result of the failure to take into account how blood glucose affected hepatic SUV.

Hepatocytes and blood dynamically exchange FDG, leading to a hepatic FDG concentration that almost matches the blood concentration [11, 12]. In fact, it has been suggested that the liver is a better location to observe the time course of blood activity in small animal studies than the heart [13]. Because blood glucose affects blood FDG concentration [11], the hepatic FDG signal is likewise reliant on blood glucose, as demonstrated earlier [14, 15], meaning that blood glucose also affects the tumor-to-liver ratio.

This study therefore aimed to investigate homogeneity and stability of liver SUV in terms of different commercial PET/CT scanners in different malignancies considering variable body normalization schemes and blood glucose concentrations.

## Materials and methods

### Patients

The study included 207 patients (mean age 55.6 ± 14.92) with four types of cancers, namely, breast, lymphoma, lung, and bone malignancies who were retrospectively retrieved from different medical centers operating F18-FDG PET/CT scanning service. All patients had undergone whole body F18-FDG PET/CT scanning using standard local imaging protocols. Patient characteristics including gender, age, and number of each type of cancer per scanner is tabulated in Table 1.

**Table 1:** Patient characteristics

Scanner	GE discovery IQ (GE healthcare)	Biograph mCT (Siemens healthineers)	uMI 550 (Shanghai united imaging healthcare Co.)	Ingenuity TF64 (Philips medical systems (Cleveland))
Number of patients	54	50	51	52
Gender				
Male	27	25	22	21
Female	27	25	29	31
Age, mean (y)	55.3 ± 14.42	55.8 ± 17.39	56.2 ± 15.68	53.3 ± 13.79
Type of cancer				
Breast	19	15	23	13
Lymphoma	14	18	10	20
Lung	13	11	11	9
Bone-mets	8	10	7	10

Patients were fasted for 6 h before commencing the imaging procedure. Extensive exercises were instructed to be avoided and injection was carried out in relaxing conditions. Data acquisition was performed with GE Discovery IQ (General Electric Healthcare), Biograph mCT (Siemens Healthineers), uMI 550 (Shanghai United Imaging Healthcare Co.), and Ingenuity TF64 (Philips Medical Systems, Cleveland) after a single intravenous injection of 194 ± 67.5 MBq of 18F-FDG (on average 2.5 MBq/kg). The scan started approximately 57.5 ± 18.5 min post-injection. Data were required with an acquisition time of 2 min/bed position in all scanners and with 5–6 bed position in United Imaging scanner, whereas 8–10 bed position in the other PET/CT systems.

Image reconstruction was applied with manufacturer standard clinical parameters. Additionally, raw data acquired with GE were reconstructed with Z-axis filter (standard) using 2 and 12 as number of iteration and subsets respectively and a full width at half maximum (FWHM) of 6.4 mm. The United Imaging data were reconstructed with smoothing function (smoothing 3) using 2 and 20 as number of iteration and subsets respectively and FWHM of 3 mm.

The Siemens images were reconstructed with Gaussian filter (TrueX + TOF) using 2 and 12 as number of iterations and subsets, respectively, and FWHM of 5 mm. The Philips images were reconstructed with iterative reconstruction algorithm filter (BLOB-OS-TF) using 3 and 33 as number of iterations and subsets, respectively, and FWHM of 4 mm.

### SUV measurements

3D MIP images were generated by merging CT and PET images with a “drag and drop” tool. The Volume Computer Assisted Reading (VCAR) software within the AW server was used to generate scaled 3D images for liver. The images were imported into GE AW workstation, and quantitative volumetric assessment was performed using the automatic hepatic VCAR tool with manual edits when needed. A ROI was drawn over the whole liver to quantify the liver SUVmax and SUVmean. Both measures have been extensively used in routine practice as well as research objectives in oncologic PET/CT scanning. All patients were absent of any abnormal uptake in the liver abnormal or metastatic disease.

Normalization of the body weight was varied including whole body weight, lean body mass, body surface area, and body mass index as follows.

Body weight (BW)

$${\text{SUV }} = { }\frac{{{\text{Concentration of activity in tumor}}\left( {{\text{MBq}}/{\text{mL}}} \right){ }}}{{{\text{Injected activity}}\left( {{\text{MBq}}} \right)/{\text{mass of patient}}\left( {\text{g}} \right)}}.$$

Lean body mass (LBM).

$${\text{LBM}}\left( \, {{\text{Female}}} \right)\, = \,{1}.0{7}\, \times \,{\text{BW - 148 }} \times \left( {{\text{BW}}/{\text{H}}} \right){2}$$

$${\text{LBM}}\left( \,{{\text{Male}}} \right)\, = \,1. 1\, \times \,{\text{BW - }}128\, \times \left( {{\text{BW}}/{\text{H}}} \right)2)$$

$${\text{SUV }} = { }\frac{{{\text{Concentration of activity in tumor}}\left( \,{{\text{MBq}}/{\text{mL}}} \right){ }}}{{{\text{Injected activity}}\left( \,{{\text{MBq}}} \right)/{\text{LBM}}}}.$$

Body surface area (BSA)

$${\text{BSA}}\left( {{\text{m}}^{2} } \right) = \sqrt {\frac{{W\left( \,{{\text{Kg}}} \right){ } \times { }H\left( \,{{\text{Cm}}} \right)}}{3600}}$$

$${\text{SUV}} = \frac{{{\text{Concentration}} {\text{of}} {\text{activity}} {\text{in}} {\text{tumor}}\left( \,{{\text{MBq}}/{\text{mL}}} \right) }}{{{\text{Injected}} {\text{activity}}\left( \,{{\text{MBq}}} \right)/{\text{BSA}}\left( {{\text{cm}}^{2} } \right)}}$$

and body mass index (BMI)

$${\text{BMI }} = {\text{ W}}/{\text{H}}^{{2}}$$

$${\text{SUV}} = \frac{{{\text{Concentration}} {\text{of}} {\text{activity}} {\text{in}} {\text{tumor}}\left( \,{{\text{MBq}}/{\text{mL}}} \right) }}{{{\text{Injected}} {\text{activity}}\left( {{\text{MBq}}} \right)/{\text{BMI}}}}.$$

Consequently, we calculate SUVmax and SUVmean with and without glucose correction for the different malignancies (breast, lymphoma, lung, and bone-mets) acquired using the different scanners. The formula used to account for blood glucose level in the SUV measurement was defined as follows:

$${\text{SUV }} \times \, \left[ {{\text{Glucose concentration }}/{\text{ Standard }}100{\text{ mg}}/{\text{dl}}} \right].$$

Table 2 summarizes comparison of the four different scanners for measurement of SUVmax and SUVmean normalized to body weight in cancer breast, lymphoma, lung, and bone-mets patients with and without glucose correction. The same is described for SUVlbm, SUVbsa, and SUVbmi in Tables 3, 4, 5, respectively.

**Table 2:** Kruskal Wallis of SUVmean and SUVmax normalized to whole body weight for patients with Breast, Lymphoma, Lung, and Bone cancers patients computed along with pair-wise Mann–Whitney test

Type of cancer				Kruskal–Wallis	GE			Siemens		United
	SUV				Siemens	United	Philips	United	Philips	Philips
Breast	Whole liver	SUVmax	Before	** < 0.0001**	**<0.000**	**<0.0001**	**0.242**	**0.137**	**0.002**	**<0.0001**
			After	** < 0.0001**	**0.080**	**0.001**	**0.659**	**0.150**	**0.015**	**<0.0001**
		SUVmean	Before	** < 0.0001**	**0.010**	**<0.0001**	**0.631**	**0.062**	**0.035**	**0.001**
			After	**0.007**	**0.662**	**0.029**	**0.173**	**0.091**	**0.089**	**0.001**
Lymphoma	Whole liver	SUVmax	Before	** < 0.0001**	**0.002**	**0.001**	**0.806**	**0.056**	**0.003**	**0.001**
			After	**0.001**	**0.451**	**<0.0001**	**0.124**	**0.007**	**0.393**	**0.001**
		SUVmean	Before	** < 0.0001**	**0.142**	**<0.0001**	**0.167**	**0.027**	**0.007**	**<0.0001**
			After	**0.003**	**0.266**	**0.002**	**0.042**	**0.012**	**0.329**	**0.002**
Lung	Whole liver	SUVmax	Before	** < 0.0001**	**0.032**	**<0.0001**	**0.764**	**0.006**	**0.141**	**0.002**
			After	**0.006**	**0.292**	**0.005**	**0.815**	**0.020**	**0.142**	**0.004**
		SUVmean	Before	**0.003**	**0.030**	**0.006**	**0.973**	**0.291**	**0.013**	**0.004**
			After	**0.150**	**0.385**	**0.077**	**0.570**	**0.573**	**0.165**	**0.044**
Bone-mets	Whole liver	SUVmax	Before	**0.003**	**0.021**	**0.005**	**0.041**	**0.081**	**0.288**	**0.011**
			After	**0.062**	**0.290**	**0.037**	**0.722**	**0.013**	**0.683**	**0.051**
		SUVmean	Before	**0.006**	**0.112**	**0.001**	**0.120**	**0.007**	**0.744**	**0.064**
			After	**0.121**	**0.736**	**0.083**	**0.859**	**0.005**	**0.624**	**0.242**

A *p* values of less than 0.05 was considered statistically signficant

**Table 3:** Kruskal Wallis of SUVmean and SUVmax normalized to lean body mass for patients with Breast, Lymphoma, Lung and Bone cancers patients computed along with pair-wise Mann–Whitney test

Type of cancer				Kruskal–Wallis	GE			Siemens		United
	SUV				Siemens	United	Philips	United	Philips	Philips
Breast	Whole liver	SUVmax	Before	**<0.0001**	**0.006**	**<0.000**	**0.227**	**<0.000**	**0.089**	**<0.000**
			After	**<0.0001**	**0.512**	**<0.0001**	**0.291**	**0.006**	**0.190**	**<0.0001**
		SUVmean	Before	**<0.0001**	**0.259**	**<0.0001**	**0.744**	**<0.0001**	**0.264**	**<0.0001**
			After	**<0.0001**	**0.662**	**0.005**	**0.068**	**0.006**	**0.332**	**<0.0001**
Lymphoma	Whole liver	SUVmax	Before	**<0.0001**	**0.039**	**0.001**	**0.100**	**0.009**	**0.009**	**<0.0001**
			After	**<0.0001**	**0.204**	**<0.0001**	**0.025**	**0.004**	**0.004**	**<0.0001**
		SUVmean	Before	**<0.0001**	**0.691**	**0.002**	**0.004**	**0.006**	**0.006**	**<0.0001**
			After	**<0.0001**	**0.074**	**0.026**	**0.006**	**0.004**	**0.004**	**<0.001**
Lung	Whole liver	SUVmax	Before	**<0.0001**	**0.022**	**<0.0001**	**0.526**	**0.002**	**0.121**	**0.001**
			After	**0.002**	**0.107**	**0.002**	**0.815**	**0.007**	**0.253**	**0.003**
		SUVmean	Before	**0.001**	**0.013**	**<0.0001**	**0.815**	**0.181**	**0.041**	**0.003**
			After	**0.029**	**0.094**	**0.014**	**0.867**	**0.360**	**0.191**	**0.014**
BONE-mets	Whole liver	SUVmax	Before	**0.001**	**0.043**	**0.008**	**0.183**	**0.004**	**0.086**	**0.001**
			After	**0.016**	**0.773**	**0.049**	**0.859**	**0.001**	**0.806**	**0.006**
		SUVmean	Before	**0.001**	**1.000**	**0.001**	**0.859**	**0.001**	**0.514**	**0.001**
			After	**0.043**	**0.211**	**0.132**	**0.790**	**0.002**	**0.624**	**0.079**

A *p* values of less than 0.05 was considered statistically signficant

**Table 4:** Kruskal Wallis of SUVmean and SUVmax normalized to body surface area for patients with Breast, Lymphoma, Lung, and Bone cancers patients computed along with pair-wise Mann–Whitney test

Type of cancer				Kruskal–Wallis	GE			Siemens		United
	SUV				Siemens	United	Philips	United	Philips	Philips
Breast	Whole liver	SUVmax	Before	**<0.0001**	**<0.0001**	**0.000**	**0.328**	**<0.0001**	**0.015**	**<0.0001**
			After	**<0.0001**	**0.216**	**<0.0001**	**0.309**	**0.011**	**0.058**	**<0.0001**
		SUVmean	Before	**<0.0001**	**0.045**	**<0.0001**	**0.803**	**0.001**	**0.073**	**<0.0001**
			After	**0.001**	**0.771**	**<0.010**	**0.048**	**0.016**	**0.089**	**<0.0001**
Lymphoma	Whole liver	SUVmax	Before	**<0.0001**	**0.043**	**0.001**	**0.294**	**0.018**	**0.001**	**<0.0001**
			After	**0.001**	**0.361**	**0.001**	**0.064**	**0.002**	**0.273**	**0.001**
		SUVmean	Before	**<0.0001**	**0.383**	**0.003**	**0.017**	**0.010**	**0.002**	**<0.0001**
			After	**0.003**	**0.062**	**0.053**	**0.025**	**0.006**	**0.247**	**0.003**
Lung	Whole liver	SUVmax	Before	**<0.0001**	**0.047**	**<0.0001**	**0.713**	**0.001**	**0.102**	**0.001**
			After	**0.003**	**0.172**	**0.004**	**0.867**	**0.005**	**0.288**	**0.003**
		SUVmean	Before	**0.001**	**0.018**	**0.001**	**0.920**	**0.091**	**0.022**	**0.003**
			After	**0.107**	**0.495**	**0.087**	**0.483**	**0.291**	**0.142**	**0.025**
Bone-mets	Whole liver	SUVmax	Before	**0.001**	**0.012**	**0.005**	**0.110**	**0.030**	**0.086**	**0.002**
			After	**0.047**	**0.564**	**0.049**	**0.929**	**0.010**	**0.624**	**0.019**
		SUVmean	Before	**0.004**	**0.336**	**0.001**	**0.328**	**0.007**	**0.744**	**0.006**
			After	**0.114**	**0.700**	**0.165**	**0.722**	**0.005**	**10.000**	**0.097**

A p values of less than 0.05 was considered statistically signficant

**Table 5:** Kruskal Wallis of SUVmean and SUVmax normalized to body mass index for patients with Breast, Lymphoma, Lung and Bone cancers patients computed along with pair-wise Mann–Whitney test

Type of cancer				Kruskal–Wallis	GE			Siemens		United
	SUV				Siemens	United	Philips	United	Philips	Philips
Breast	Whole liver	SUVmax	Before	**<0.000**	**<0.0001**	**<0.0001**	**0.552**	**0.594**	**0.001**	**0.001**
			After	**0.002**	**0.094**	**0.017**	**0.367**	**0.364**	**0.015**	**0.000**
		SUVmean	Before	**0.003**	**0.032**	**0.004**	**0.833**	**0.332**	**0.020**	**0.005**
			After	**0.025**	**0.536**	**0.211**	**0.081**	**0.273**	**0.033**	**0.004**
Lymphoma	Whole liver	SUVmax	Before	**0.002**	**0.049**	**0.002**	**0.916**	**0.079**	**0.041**	**0.002**
			After	**0.008**	**0.905**	**0.001**	**0.208**	**0.035**	**0.345**	**0.003**
		SUVmean	Before	**0.004**	**0.691**	**0.002**	**0.151**	**0.132**	**0.120**	**0.000**
			After	**0.020**	**0.383**	**0.026**	**0.093**	**0.035**	**0.428**	**0.006**
Lung	Whole liver	SUVmax	Before	**0.001**	**0.172**	**0.001**	**0.713**	**0.002**	**0.072**	**0.002**
			After	**0.021**	**0.495**	**0.030**	**0.570**	**0.011**	**0.221**	**0.011**
		SUVmean	Before	**0.012**	**0.239**	**0.026**	**0.483**	**0.181**	**0.041**	**0.003**
			After	**0.303**	**0.951**	**0.505**	**0.333**	**0.360**	**0.253**	**0.044**
Bone-mets	Whole liver	SUVmax	Before	**0.003**	**0.009**	**0.003**	**0.214**	**0.101**	**0.142**	**0.015**
			After	**0.091**	**0.441**	**0.021**	**0.859**	**0.050**	**0.514**	**0.064**
		SUVmean	Before	**0.030**	**0.211**	**0.002**	**0.722**	**0.081**	**0.624**	**0.040**
			After	**0.222**	**0.700**	**0.064**	**0.594**	**0.039**	**0.744**	**0.205**

A p values of less than 0.05 was considered statistically signficant

### Statistical analysis

Statistical analysis was performed using dedicated software (IBM SPSS Statistics for Windows, v. 23.0.0, Armonk, NY). The comparison of the four different scanners for measurement of SUVmax and SUVmean normalized to (body weight, lean body mass, body surface area, and body mass index) in cancer breast, lymphoma, lung, and bone-mets patients with and without glucose correction and characteristics was performed with either the Mann–Whitney *U* test (two independent samples) or the Kruskal–Wallis test (three or more independent samples). A *p* value less than 0.05 was considered statistically significant.

## Results

**Fig. 1:**
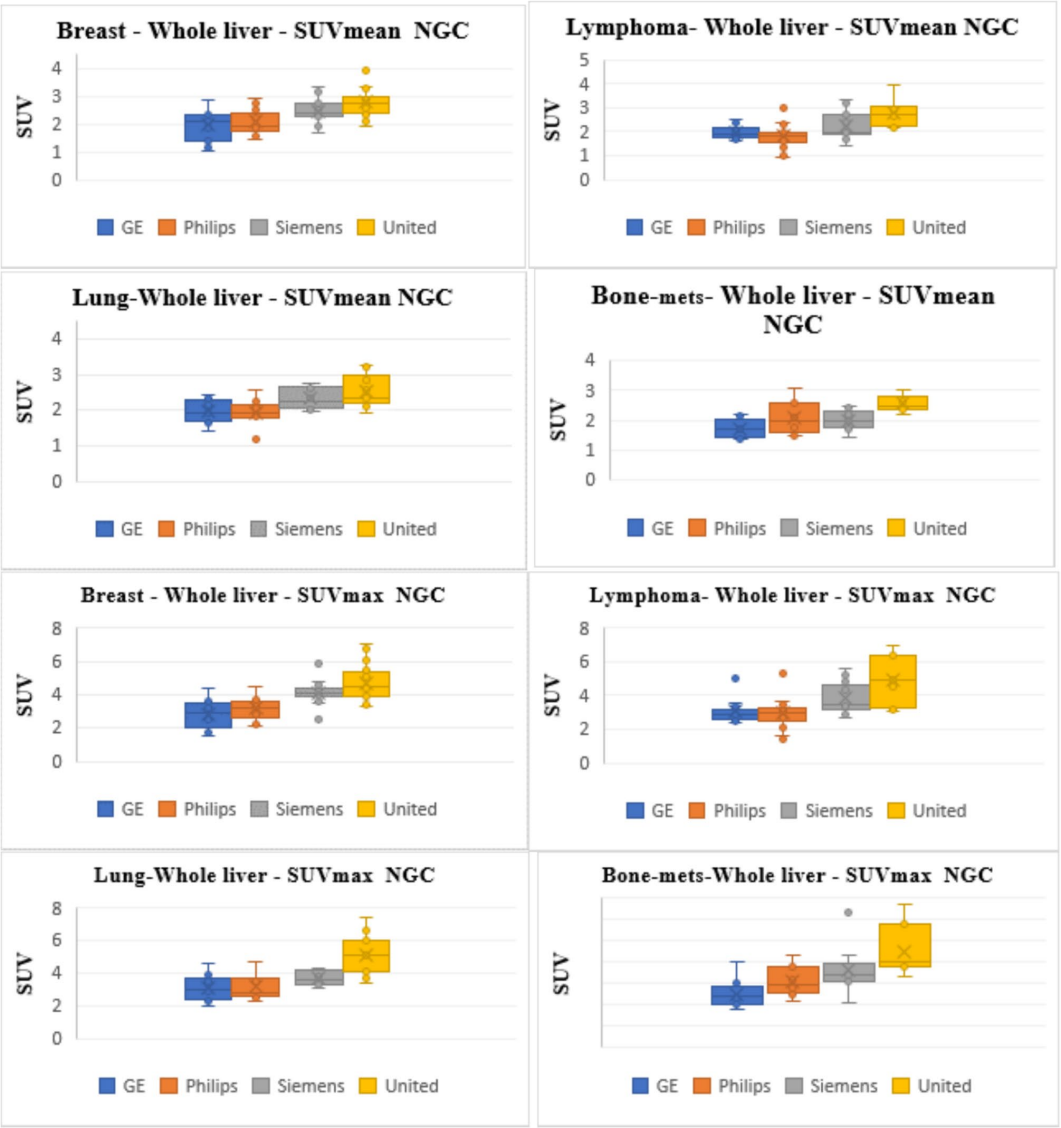
Boxplots showing the median and quartiles of SUVmean (upper panel) and SUVmax (lower panel) measured using the four different scanners (i.e., GE, Philips, Siemens, and United Imaging) for 4 different types of malignancies (i.e., breast, lymphoma, lung, and bone). SUV measured using whole body weight normalization (i.e., SUVbw). Data presented are not corrected for glucose levels

**Fig. 2:**
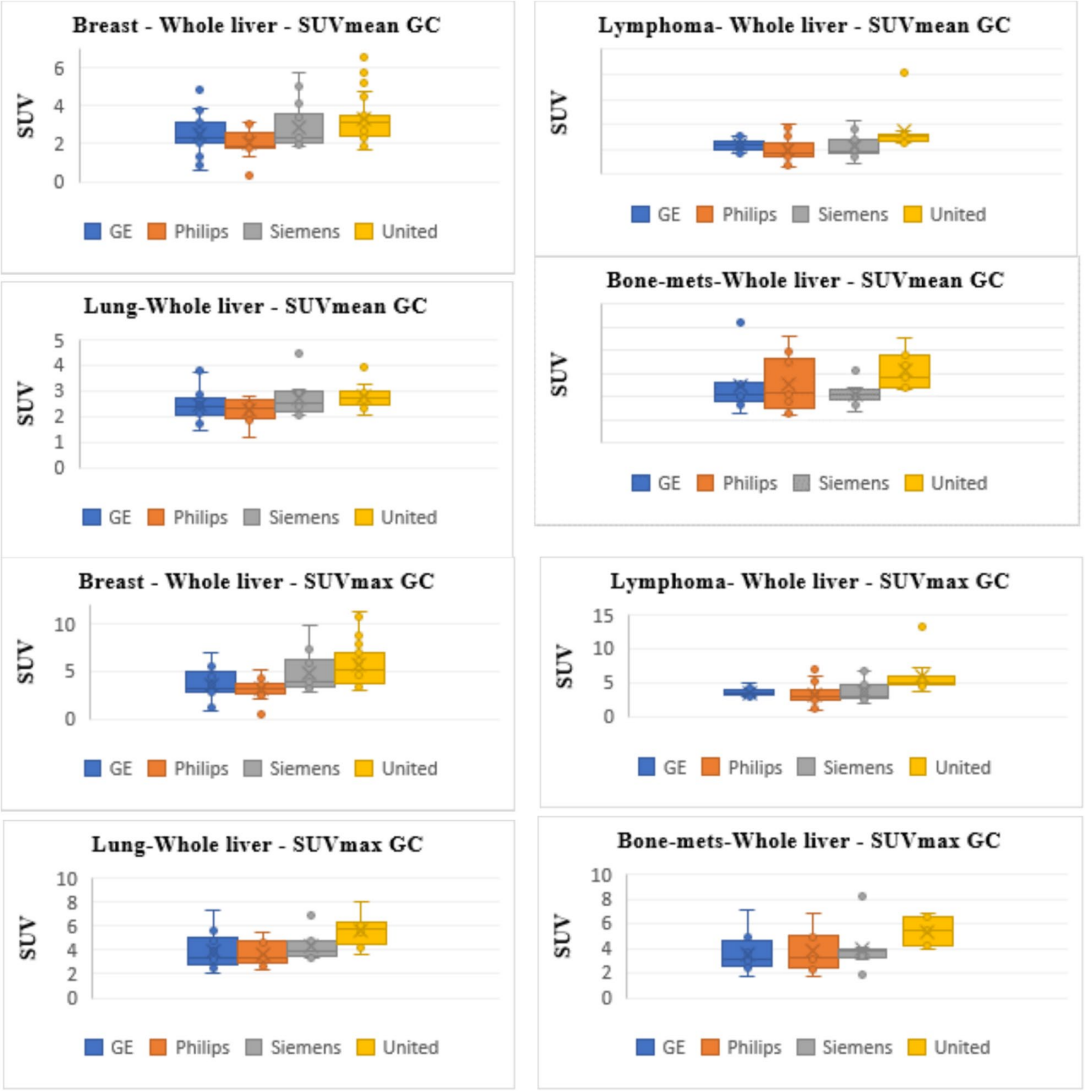
Boxplots showing the median and quartiles of SUVmean (upper panel) and SUVmax (lower panel) measured using the four different scanners (i.e., GE, Philips, Siemens, and United Imaging) for 4 different types of malignancies (i.e., breast, lymphoma, lung, and bone). SUV measured using whole body weight normalization (i.e., SUVbw). Data presented are corrected for glucose levels

**Fig. 3:**
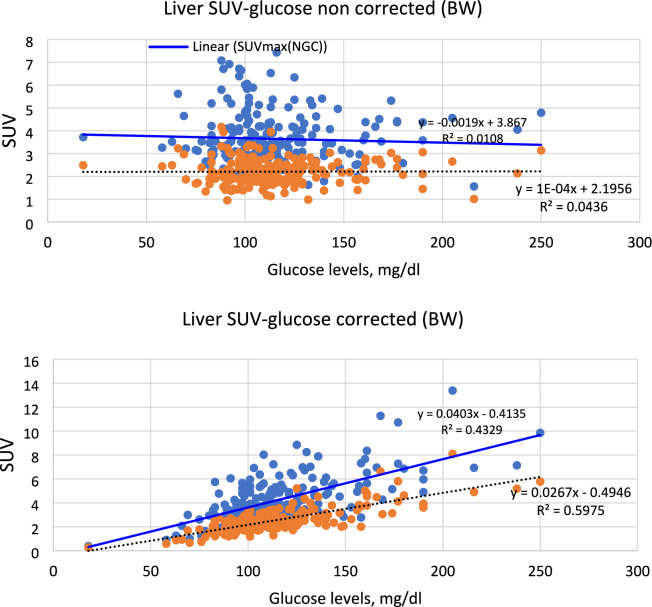
correlation between liver SUVmax and SUVmean with blood glucose level for non-corrected (upper panel) and glucose corrected (lower panel) measured using the four different scanners (i.e., GE, Philips, Siemens, and United Imaging) for 4 different types of malignancies (i.e., breast, lymphoma, lung, and bone). SUV measured using whole body weight normalization (i.e., SUVbw). Solid line represents the SUVmax whereas the dashed line represented SUVmean

Comparisons of the four different scanners for measurement of SUVmax and SUVmean normalized to body weight in cancer breast, lymphoma, lung, and bone-mets patients with and without glucose correction are illustrated in Figs. 1 and 2. Normalizing SUV to other body weights including lean body mass, body surface area, and body mass index are demonstrated in the supplementary material file referred to figure S1-2, S4-5, S7-8, respectively. The correlation of the SUVmax and SUVmean to the glucose level is described in Fig. 3 for body weight normalization but in S3, S6, and S9 for lean body mass, body surface area, and body mass index, respectively.

The statistical significance (i.e., *p* value) among the 4 scanners and the 4 different malignancies is summarized in Tables 2, 3, 4, 5 for SUVmax and SUVmean normalized to whole body weight, lean body mass, body surface area, and body mass index including data before and after glucose correction. Additionally, the non-parametric Mann–Whitney pair-wise comparison is demonstrated in Tables 2, 3, 4, 5 for the 4 scanners included in the study.

### SUVbw

In SUVbw, there was a statistically significant difference among the 4 different scanners for the 4 neoplasms including those measurements corrected and non-corrected for glucose. Nonetheless, SUVmax and SUVmean in bone-mets as well as SUVmean in lung patients were not statistically different among scanners especially for data corrected for glucose levels (*p = *0.062, 0.121, and 0.150 respectively).

For pair-wise comparison among the different scanners in lung cancer, the SUVmean of the GE was not significantly different from the other 3 scanners (all *p > *0.05) and Siemens was also not significantly different from United and Philips measurements. However, United and Philips were statistically different (*p = *0.044). Similarly, the pair-wise comparison of the SUVmax measured by GE for bone-mets cancer revealed a non-significant difference between GE versus Siemens and Philips (*p = *0.290 and 0.722, respectively). Moreover, Philips was also not significantly different from Siemens and United Imaging (*p = *0.683 and 0.051, respectively).

SUVmean of the bone-mets cancer patients provided a pair-wise comparison that is not statistically significant between GE and the other systems, including Siemens, United Imaging, and Philips (*p = *0.736, 0.083, and 0.859, respectively) for data corrected for glucose levels. Moreover, Philips was not significantly different from Siemens and United Imaging (0.624, and 0.242 respectively).

### SUVlbm

In measurements of SUV corrected for LBM, all comparisons using the Kruskal–Wallis test was significant among the 4 different scanners for the different malignancies (*p* < 0.05), as shown in Table 3. However, there were some non-significant differences in pair-wise comparisons notably in bone-mets and lung cancers, Table 3. In lung patients, there was no significant differences in Philips in comparison to GE and Siemens (both, *p > *0.05) for data corrected and not corrected for glucose levels. Also, GE and Siemens were not significantly different (*p = *0.094) after glucose correction. In bone-mets patients, the GE system revealed results that are not significantly different from the other 3 scanners for data corrected for glucose levels. Philips system also provided results that were not statistically different from Siemens and United Imaging (*p = *0.624 and 0.079, respectively).

### SUVbsa

In SUV normalized to BSA, the only non-significant difference revealed among scanners in the measurements of SUVmean obtained from lung and bone-mets (*p = *0.107 and 0.114) both for data corrected for glucose levels, Table 4. In pair-wise comparisons of lung patients, all pairs of scanners were not significantly different for SUVmean corrected for glucose (*p > *0.05) except Philips and United Imaging were statistically different (*p = *0.025). In pair-wise comparison of bone-mets patients, all pair-wise comparisons were not statistically different for measurements of SUVmean except United Imaging versus GE and Siemens (*p = *0.005) for data corrected for glucose levels, Table 4.

### SUVbmi

In measurements of SUV normalized to body mass index, SUVmean of lung and bone-mets cancers as well as SUVmax of bone-mets showed a non-significant differences among the 4 different scanning systems (*p = *0.303, 0.091, and 0.222, respectively) for data corrected for glucose levels, Table 5. In pair-wise testing, there were multiple comparisons that showed also a non-significant differences notably in breast cancer patients in measurements of SUVmean after glucose correction (*p > *0.05) except Philips versus Siemens and United Imaging (*p = *0.033 and 0.004 respectively). In lung cancers, all pair-wise comparisons of the SUVmean corrected for glucose levels among scanners were not significant except United Imaging vs Philips (*p = *0.044). In bone-mets patients, all pair-wise comparisons of SUVmax corrected for glucose were similarly not significant except GE and United Imaging (*p = *0.021). SUVmean corrected for glucose in bone-mets cancers were also not significant among scanners except Siemens vs United Imaging (*p = *0.039).

### Liver SUV and blood glucose

As shown in Fig. 3 and Table 6, SUVmax normalized to BW showed a non-significant correlation with blood glucose (*r* = 0.104, *p *= 0.136) but significant with SUVmean (* r = 0.209 *, *p* = 0.002). However, after glucose correction, a strong correlation was obtained (*r* = 0.658 and 0.773, respectively, *p* < 0.0001). In LBM, there was also a non-significant correlation (*r* = 0.043 and 0.136, *p* > 0.05) that become significant after glucose correction (*r* = 0.630 and 0.756, both *p* < 0.0001) for SUVmax and SUVmean, respectively.

**Table 6:** Correlation results of the liver SUVmean and SUVmax before and after glucose correction with different body weight normalizations

Normalization	SUV	Glucose correction	*r*	*p* value
BW	Max	Before	0.104	0.136
		After	0.658	< 0.0001
	Mean	Before	0.209	0.002
		After	0.773	< 0.0001
LBM	Max	Before	0.043	0.537
		After	0.630	< 0.0001
	Mean	Before	0.136	0.051
		After	0.756	< 0.0001
BMI	Max	Before	0.089	0.200
		After	0.650	< 0.0001
	Mean	Before	0.165	0.017
		After	0.742	< 0.0001
BSA	Max	Before	0.073	0.296
		After	0.671	< 0.0001
	Mean	Before	0.169	0.015
		After	0.783	< 0.0001

A *p* values of less than 0.05 was considered statistically signficant

In normalization to BMI, the same pattern was observed where a non-significant and poor correlation of SUVmax and SUVmean versus blood glucose (*r* = 0.089 and 0.165, *p = *0.200 and 0.017, respectively). The same was obtained in BSA normalization, and a non-significant and poor correlation was found before (*r* = 0.073 and 0.169, *p = *0.296 and 0.015) that significantly improved after glucose adjustment (*r* = 0.671 and 0.783, both *p* < 0.0001).

## Discussion

Determination of SUV liver uptake has been very instrumental in many quantitative aspects in PET/CT imaging data evaluation. The use of liver SUV enjoys stable measurements and generally used as background tissues when malignant uptake is being observed. Moreover, normalizing data to liver uptake provides reference measurements to evaluate changes or compare different tissues uptakes. Recently, our group showed that liver has more stable and reduced variations when compared to aorta and muscle uptake in patients with lymphoma [16]. The liver SUV measurements were also shown to be influenced by the weight normalization index [16].

Blood glucose level can affect SUV measurements, because FDG has a molecular pathway similar to the mechanism by which glucose is metabolized within cells. The enzyme hexokinase phosphorylates the glucose once taken up by malignant cells creating FDG-6-phosphate that is trapped in the cell [17]. The FDG is in competition with glucose, because hexokinase also phosphorylates glucose to form glucose-6-phosphate [18].

Glucose and 18F-FDG compete to enter the cells using the same glucose transporters. High blood glucose levels (GL) reduce 18F-FDG uptake in tissues by competitive inhibition altering the biodistribution of FDG. The reason for fasting prior to the start of the PET/CT study is to achieve fairly low values of glucose levels to enhance target-to-background and improve image contrast [19, 20]. The end effect is that the high blood glucose serves to reduce SUV measurements due to competitive inhibition of FDG uptake.

The results of studies assessing the value of accounting for blood glucose have been inconsistent. Normalizing SUV by blood glucose has been beneficial in some cases [21–23], not useful in others [24–26], and decreasing the repeatability of SUV readings in the other cases [27, 28]. One study suggests that for GL < 200 mg/dl, correction of blood glucose is not necessary [29]. Patients with normal blood glucose levels are therefore more likely to benefit from glucose correction, whereas those with high levels are less likely to require it [29, 30].

Some national and international guidelines on the use of PET/CT is to avoid correcting SUVs for blood glucose if the levels are within the reference range (< 200 mg/dl) [31]. In a PET/CT protocol for multicenter trials in The Netherlands, it is recommended that patients are to be rescheduled only if blood glucose is above twice the normal concentration [32].

Because of variations in SUVs, tumor to reference ratios (tumor/liver or tumor/blood pool ratios) or SUV normalized to LBM are used to better assess response to treatments in oncologic cases. As reference, SUVs should be measured from the normal parts of the liver. In cases with diffuse involvement of the liver with various diseases such as metastases or hepatitis, liver should not be used as normal reference. In the present study, the whole liver was segmented and processed for further analysis after ensuring the normality of the liver tissues. One reportstated that tumor FDG uptake (simulated using brain tissues) cannot be factored to liver SUV because of the effects of blood glucose apart from any influence of hepatic fat [33].

Despite liver uptake might be a stable and reference tissue in interpretation of PET/CT, the present study showed significant differences among scanners in measurements of SUVmax and SUVmean in individual cancers. The United Imaging and Siemens scanners have shown consistent increase in SUV when compared to those values obtained with GE and Siemens. However, after glucose correction, a more stable and less deviation in part was observed in SUV measurements among the scanners.

Three out of the four different types of normalizations used for SUV measurements (namely, body weight, body surface area, and body mass index) have been efficient and showed insignificant differences in the SUVmean of bone-mets and lung patients after glucose correction. Lean body mass-normalized SUV showed significant differences among scanners in all cancers. However, pair-wise comparison showed some benefits especially in SUVmean after glucose correction (except Siemens vs United, *p = *0.002). It appeared that SUVmean was frequently more performant than SUVmax. However, SUVmax was beneficial in cases of bone-mets when body mass index was used in SUV normalization.

Despite the relatively encouraging results reported above on the improved homogeneity of SUV measurements, certain variations were still seen among scanners following the use of glucose adjustment in a given cancer. These differences were noteworthy and regularly observed in United vs the other scanners including Philips, GE, and Siemens. With a close look into Figs. 1 and 2, one can observe the consistent higher values produced by the United Imaging versus the other PET/CT systems.

As outlined earlier, liver SUV is used as a reference to better assess response to treatments in patients with various tumors. SUVmean or SUVmax of the liver is used although SUVmean is more stable due to a slight heterogeneity in FDG distribution in normal liver that impacts the stability of the SUVmax [34, 35]. This is largely consistent with the findings presented in this study. The SUVmean in bone-mets and lung cancer was found to be more successful after glucose correction in providing more uniformity among the different types of PET/CT systems.

Knowing baseline SUV variations of patients prior to treatment makes an important contribution in determining pathological F18-FDG involvement areas and in evaluating tumor response. FDG brain uptake is influenced by glucose levels in an inverse relationship. Correction of the glucose concentration in some areas of the brain, such as cerebellum, basal ganglia, and frontal cortex, could provide SUV measurements independent of blood glucose [33]. However, when brain SUVmax was divided by liver SUVmax, it showed almost no relationship with blood glucose, regardless of whether patients had hepatic steatosis [33]

It is unclear whether glucose normalization improves diagnosis accuracy or treatment response monitoring of malignant tumors [36]. One study has reported the application of the SUV glucose corrected in lung nodules [30]. The results did not support the use of this correction to differentiate lung nodules; this was most likely caused by the small range of glucose levels adopted in the study (< 150 mg/dl) [37].

Liver SUVmax and SUVmean were found to poorly correlate with blood glucose before adjustment for glucose correction in all body weight normalization methods. The values were consistent with meta-analysis performed on a large patient population [29]. However, the correlation has significantly been improved after glucose correction. SUVmean consistently had higher correlation than SUVmax. Moreover, the correlation was increasing in this ascending order BMI, LBM, BW, and BSA noting that the values were not significantly different among the 4 methods (*r* = 0.742–0.783).

Therefore, in particular situations when liver SUV needs to be correlated with blood glucose levels, important considerations need to be carried out in terms of weight normalization as well as blood glucose concentration. The highest correlation obtained was in SUVmean normalized to whole body weight. Nevertheless, these adjustments need to be considered on an individual pathological basis. This information may also contribute to design standardized protocols for liver glucose correction.

Data analysis presented in this study provides encouraging results to continue investigating glucose correction-based liver SUV on individual tumors. The lung malignancies and bone-metastasis patients were the only two conditions who predominantly showed harmonization among scanners when glucose correction was attempted. It is therefore highly warranted to conduct future studies carefully examining liver–glucose correction and the influence of this measure on the clinical question using large population data with good sampling statistics.

In lung cancer, chronic inflammation or certain lung cancer subtypes might alter glucose uptake in the liver [37–39]. Applying a liver-specific glucose correction factor could adjust for these metabolic fluctuations and improve detection accuracy. Lung tumors often exhibit high metabolic heterogeneity, meaning that different parts of the tumor absorb glucose at varying rates [40–43]. This can further complicate accurate SUV interpretation. Liver tissue, although not devoid of heterogeneity, generally displays more uniform glucose uptake, making it a potentially more reliable reference for correction.

Accurate and consistent SUV measurements are crucial for assessing tumor metabolism, monitoring treatment response, and making informed decisions about patient management. Liver SUV normalization could serve as a standardized reference point for evaluating changes in tumor activity, thereby aiding in the early detection of treatment response or disease progression.

The findings of this study could contribute to the development of standardized reference ranges for liver SUV in different patient populations. These reference ranges could be used to identify outliers and ensure that SUV measurements are within expected limits, thereby improving the accuracy of PET/CT interpretations.

We recently showed that the use of the liver SUV as reference was beneficial in lymphoma patients in which normalizing SUV to BSA and BMI resulted in the lowest variation, whereas using lean body mass body weight led to higher variability [16]. In the same report, SUVmax normalized to James' LBM formula and SUVmean normalized to Janma’s LBM formula and BSA showed significant independence from body weight. Those findings when combined with data presented here would provide a foundation and some guidance on the selection of weight normalization and its influence on SUV measurements.

Several precautions, however, should be considered in terms of drugs that might impact liver metabolism, presence of liver lesions or other patient-related factors that impact liver function and/or glycolytic pathways. Standardized protocols that include liver SUV normalization would help reduce inter-scanner variability and improve the reproducibility of SUV measurements across different institutions and patient populations.

This study is retrospective, a reason that introduced certain limitations in data collection. For instance, there were a higher number of female patients compared to male patients in some imaging systems. Additionally, the prevalence of different types of cancers varies, with some cancers being more common than others. Variability is also observed in patient body weight, height, age, injection dose, and the distribution of patients across different scanners.

Furthermore, the study involves a heterogeneous patient population with varying characteristics, including differences in body composition, underlying health conditions, and metabolic profiles. These variations can influence SUV measurements and introduce variability when comparing data across different scanners. The normalization of SUV to different body metrics (e.g., body weight, lean body mass, body surface area, and BMI) aims to mitigate some of these differences, but may not fully account for the impact of these patient-specific factors.

While the present and previous research efforts have scratched the surface of using the liver as reference tissue in PET/CT imaging, there is still large room for other work to investigate different approaches for glucose correction, such as the use of individualized versus population-based correction factors, evaluate the performance of various glucose correction models (e.g., linear, exponential) in different tumor types and their impact on liver SUV normalization and assess the feasibility and utility of incorporating real-time glucose monitoring during PET/CT imaging. Moreover, it might also be advantageous to follow patients over time to evaluate the temporal changes in liver SUV and its relationship with disease progression, treatment response, and other clinical outcomes. This would certainly be able to display the impact of liver SUV normalization on the interpretation of serial PET/CT scans and its potential to improve response assessment and patient management.

It is also stimulating nowadays to develop advanced computational algorithms or machine learning models to automate and optimize the process of liver SUV quantification and normalization as well as evaluate the performance of these techniques in improving the consistency, reliability, and clinical interpretation of liver SUV measurements.

In clinical trials when harmonization among systems is of key importance, it might be useful to account for glucose levels in the calculations of the liver SUVmean. However, the present study did not split patients into different categories of glucose concentrations that should be considered in future studies. Taken together, further research efforts need to be carried out focusing on specific malignant disorders investigating PET lesions normalized to liver tissues-corrected for blood glucose levels.

## Conclusions

Further research on liver glucose adjustment in individual tumors is necessary, but it may be influenced by the imaging system, SUV normalization (i.e., body weight, lean body mass, body surface area, and body mass index), and whether measurements are made on SUVmean versus SUVmax. Accounting for those parameters, this would provide a more homogenized liver SUV values. Correction of the SUV for blood glucose would permit an improvement of the correlation of the liver SUV and blood glucose concentration. This might be useful if the aim is to find an imaging biomarkers that reflects patients’ blood glucose level. Normalization to body weight and body surface area would provide the highest correlation. SUVmean remains useful and stable metric when liver is corrected for blood glucose levels in comparison to SUVmax.

## Supplementary Information

Below is the link to the electronic supplementary material.

Supplementary file1 (DOCX 436 KB)

## Data Availability

Data are available upon reasonable request from the corresponding author.
