# Correction: Estimation of liver standardized uptake value in F18-FDG PET/CT scanning: impact of different malignancies, blood glucose level, body weight normalization, and imaging systems

**DOI:** 10.1007/s12149-025-02019-6

**Published:** 2025-02-02

**Authors:** Mohamed S. Abd-Elkader, Sherif M. Elmaghraby, Mohamed A. Abdel-Mohsen, Magdy M. Khalil

**Affiliations:** 1 Department of Physics, Faculty of Science, Helwan University, Cairo, Egypt; 2 Nuclear Medicine and Radiation Oncology Department (NEMROK), Cairo University Hospitals, Faculty of Medicine, Cairo University, Cairo, Egypt; 3 School of Applied Health Sciences, Badr University in Cairo (BUC), Cairo, Egypt; 4 Medical Biophysics, Department of Physics, Faculty of Science, Helwan University, Cairo, Egypt

**Correction: Annals of Nuclear Medicine** 10.1007/s12149-024-01985-7

In this article, the ‘Conclusion’ in the “Abstract” should have read ‘Liver glucose correction needs further investigations in individual tumors but could be potentially affected by whether measurements are made on SUVmean versus SUVmax, body weight normalization, as well as the imaging system. As such, selection of normalization to body weight method should be carefully selected before clinical adoption. SUVmean proves to be useful and stable metric when liver is corrected for blood glucose levels’.

