# Supplementary material for: Estimation of liver standardized uptake value in F18-FDG PET/CT scanning: impact of different malignancies, blood glucose level, body weight normalization, and imaging systems
Source: Ann Nucl Med. 2024 Oct 16;39(2):176–88. doi: 10.1007/s12149-024-01985-7 (PMC11799010; doi:10.1007/s12149-024-01985-7)
Supplement: Supplementary file 1 — Supplementary file1 (DOCX 436 KB) [file 12149_2024_1985_MOESM1_ESM.docx]

| **Supplement** |
| --- |
| 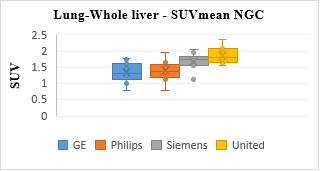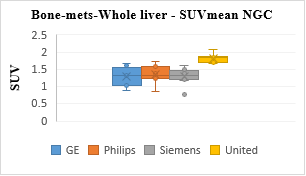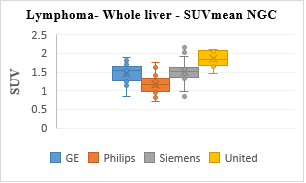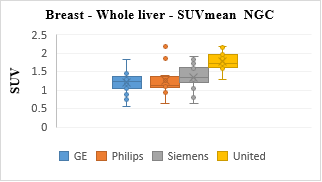 |
| 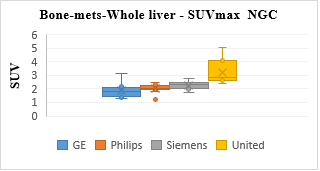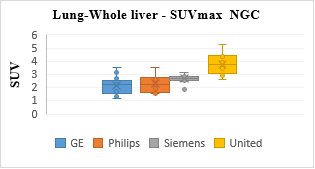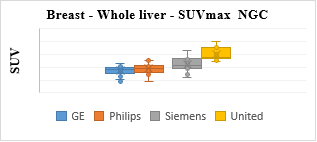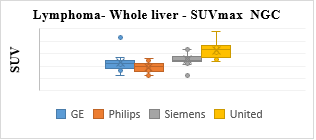SUVmax [4 figures] |
| **Figure S1.** Box plots showing the median and quartiles of SUVmean (upper panel) and SUVmax (lower panel) measured using the four different scanners (i.e. GE, Philips, Siemens, and United Imaging) for 4 different types of malignancies (i.e. Breast, Lymphoma, Lung and Bone-mets ). SUV measured using **Lean body weight normalization** (i.e. SUVlbm). Data presented are not corrected for glucose levels. |
| **Supplement** |
| 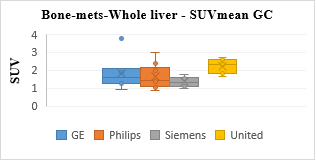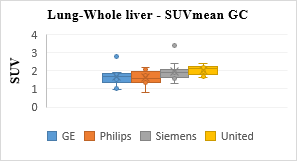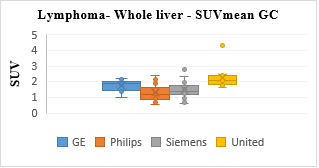  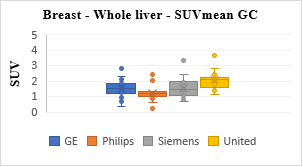 |
| 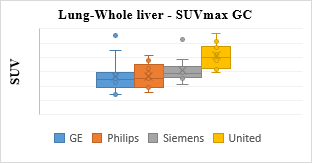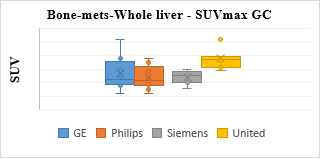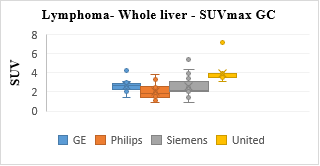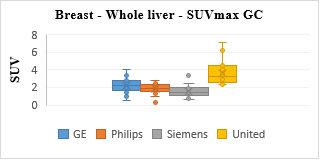 |
| **Figure S2.** Box plots showing the median and quartiles of SUVmean (upper panel) and SUVmax (lower panel) measured using the four different scanners (i.e. GE, Philips, Siemens, and United Imaging) for 4 different types of malignancies (i.e. Breast, Lymphoma, Lung and Bone-mets ). SUV measured using **lean body weight normalization** (i.e. SUVlbm). Data presented are corrected for glucose levels. |
|  |
|  |
| **Figure S3.** Relation between blood glucose and liver SUVmax and SUVmean non corrected (upper panel) and glucose corrected (lower panel) measured using the four different scanners (i.e. GE, Philips, Siemens, and United Imaging) for 4 different types of malignancies (i.e. Breast, Lymphoma, Lung and Bone-mets ). SUV measured using whole body weight normalization (i.e. **SUVlbm**). |

| **Supplement** |
| --- |
| 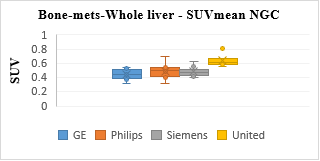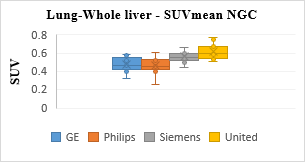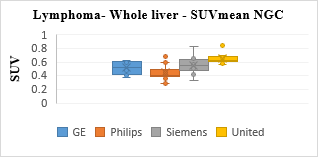  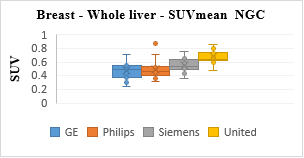 |
| 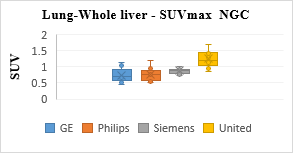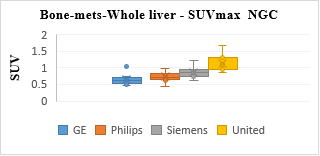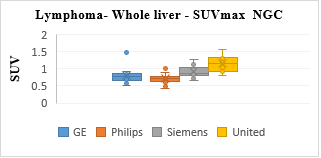SUVmax [4 figures]  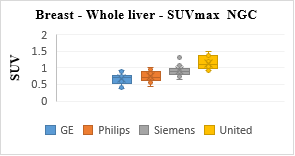 |
| **Figure S4.** Bar plots showing the median and quartiles of SUVmean (upper panel) and SUVmax (lower panel) measured using the four different scanners (i.e. GE, Philips, Siemens, and United Imaging) for 4 different types of malignancies (i.e. Breast, Lymphoma, Lung and Bone-mets ). SUV measured using **body surface area normalization** (i.e. SUVbsa). Data presented are not-corrected for glucose levels. |

| **Supplement** |
| --- |
| 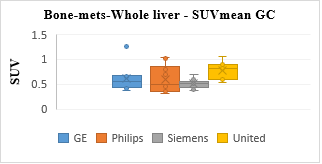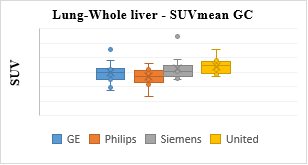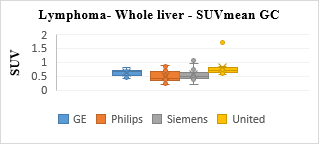  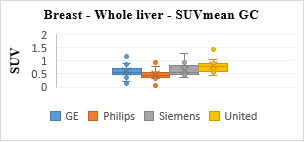 |
| 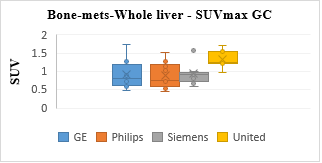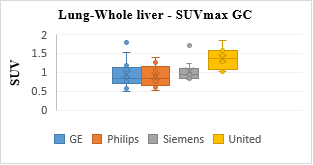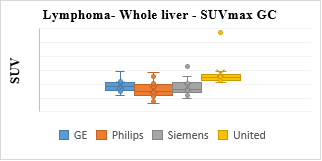  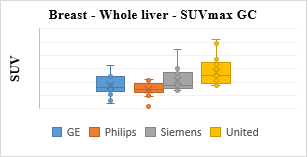 |
| **Figure S5.** Bar plots showing the median and quartiles of SUVmean (upper panel) and SUVmax (lower panel) measured using the four different scanners (i.e. GE, Philips, Siemens, and United Imaging) for 4 different types of malignancies (i.e. Breast, Lymphoma, Lung and Bone-mets ). SUV measured using **body surface area normalization** (i.e. SUVbsa). Data presented are not-corrected for glucose levels. Data presented are corrected for glucose levels. |
|  |
|  |
| **Figure S6.** Relation between (SUVmax and SUVmean) for liver with blood glucose level non corrected (upper panel) and (SUVmax and SUVmean) for liver with blood glucose level corrected (lower panel) measured using the four different scanners (i.e. GE, Philips, Siemens, and United Imaging) for 4 different types of malignancies (i.e. Breast, Lymphoma, Lung and Bone-mets ). SUV measured using whole body weight normalization (i.e. **SUVbsa**). |

| **Supplement** |  |
| --- | --- |
| 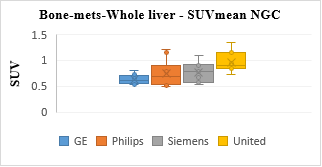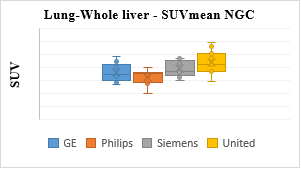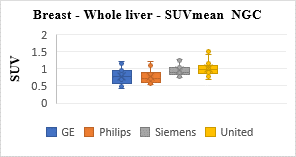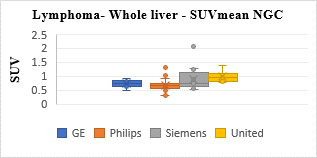 |  |
| 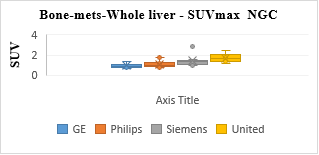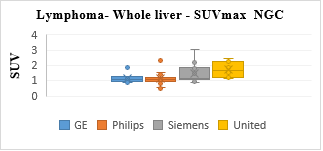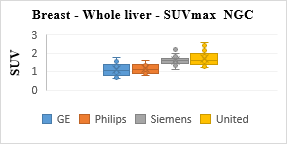  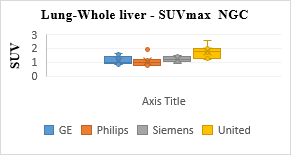 |  |
| **Figure S7**. Bar plots showing the median and quartiles of SUVmean (upper panel) and SUVmax (lower panel) measured using the four different scanners (i.e. GE, Philips, Siemens, and United Imaging) for 4 different types of malignancies (i.e. Breast, Lymphoma, Lung and Bone-mets ). SUV measured using **body mass index normalization** (i.e. SUVbmi). Data presented are not-corrected for glucose levels. |  |
| 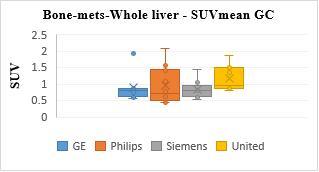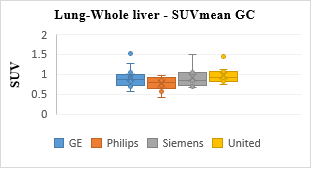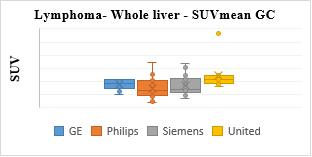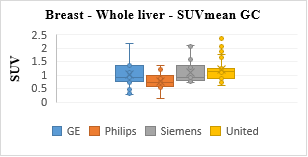 | |
| 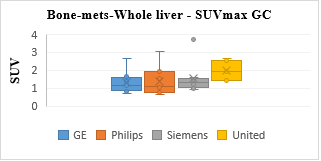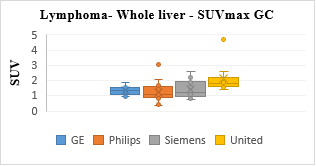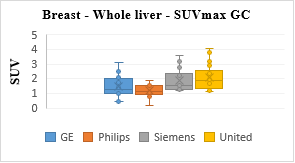  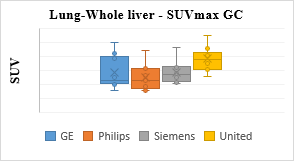 | |
| **Figure S8**. Bar plots showing the median and quartiles of SUVmean (upper panel) and SUVmax (lower panel) measured using the four different scanners (i.e. GE, Philips, Siemens, and United Imaging) for 4 different types of malignancies (i.e. Breast, Lymphoma, Lung and Bone-mets ). SUV measured using **body mass index normalization** (i.e. SUVbmi). Data presented are corrected for glucose levels. | |

|  |
| --- |
|  |
| **Figure S9.** Relation between (SUVmax and SUVmean) for liver with blood glucose level non corrected (upper panel) and (SUVmax and SUVmean) for liver with blood glucose level corrected (lower panel) measured using the four different scanners (i.e. GE, Philips, Siemens, and United Imaging) for 4 different types of malignancies (i.e. Breast, Lymphoma, Lung and Bone-mets ). SUV measured using body mass index normalization (i.e. **SUVbmi**). |
